# Numerical and Experimental Evaluation of High-Frequency Unfocused Polymer Transducer Arrays

**DOI:** 10.3390/s18061908

**Published:** 2018-06-12

**Authors:** Anowarul Habib, Sanat Wagle, Adit Decharat, Frank Melandsø

**Affiliations:** Department of Physics and Technology, UiT The Arctic University of Norway, 9037 Tromsø, Norway; sanat.wagle@uit.no (S.W.); adit.decharat@uit.no (A.D.); frank.melandso@uit.no (F.M.)

**Keywords:** polyvinylidenefluoride, needle hydrophone, acoustic pressure field, high-frequency P(VDF-TrFE) transducers, polyethyleneimine, k-Wave

## Abstract

High-frequency unfocused polymer array transducers are developed using an adhesive-free layer-by-layer assembly method. The current paper focuses on experimental and numerical methods for measuring the acoustic performance of these types of array transducers. Two different types of numerical approaches were used to simulate the transducer performance, including a finite element method (FEM) study of the transducer response done in COMSOL 5.2a Multiphysics, and modeling of the excited ultrasonic pressure fields using the open source software k-Wave 1.2.1. The experimental characterization also involves two methods (narrow and broadband pulses), which are measurements of the acoustic reflections picked up by the transducer elements. Later on, measurements were undertaken of the ultrasonic pressure fields in a water-scanning tank using a hydrophone system. Ultrasonic pressure field measurements were visualized at various distances from the transducer surface and compared with the numerical findings.

## 1. Introduction

Quantitative acoustic imaging and characterization using ultrasonic transducers has emerged within many fields related to nondestructive testing (NDT) and medical imaging. These include the noninvasive micro-structural characterization of materials, characterization of surface and subsurface mechanical properties of piezoelectric materials, structural health monitoring of composite structures, surface defects on polymer circuit, and studies of anisotropic phonon propagations [1,2,3,4,5,6,7,8,9]. A typical situation for transducer design is that a trade-off has to be made between production complexity, bandwidth, and sensitivity [10]. Although the electromechanical coupling coefficient of ferroelectric polyvinylidenefluoride (PVDF) and its copolymer PVDF trifluoroethylene [P(VDF-TrFE)] are lower than piezoceramic materials, polymer transducers have been successfully employed for various high-frequency ultrasound imaging in biological applications [11,12]. The application of P(VDF-TrFE) copolymer transducers has also been broadened from hydrophone fabrication to ultrasonic imaging of the microstructures of tissue, in, for example, dermatology, ophthalmology, and bio-microscopy [13,14,15,16,17]. At the same time, these piezoelectric polymers are very suitable materials for achieving high frequencies with a large bandwidth for ultrasonic sensors and transducers [18,19,20,21,22,23]. The flexible piezoelectric film of PVDF and its copolymer are fabricated by techniques such as spin coating, hot pressing, stamping, and spraying on a flat substrate [11,24,25,26,27,28,29]. Polymer-based transducers also typically provide a much better acoustic match with commonly used materials like water, human tissue, and polymers than transducers made from inorganic piezoelectric materials [24,25]. At the same time, the materials are flexible to some extent, and relatively easy to process. A large impedance mismatch, as for example between sapphire and water for a ZnO transducer, will typically require one or several match layers in front of the transducer for broad-band applications, which again will diminish the sound transmission and increase the geometrical aberration for both focused and unfocused transducer beams [4,11,30,31]. One the other hand, polymer-based materials have several disadvantages compared to inorganic transfer materials. These includes, for example, enhanced temperature variation of physical properties, lower processing temperatures, lower piezoelectric coupling, and higher mechanical and electrical loss [32].

In this paper, we focus on the ultrasonic field generated by a high-frequency P(VDF-TrFE) unfocused transducers fabricated using a layer-by-layer deposition method on a polyethyleneimine (PEI) substrate acting as the transducer backing. It should be noted that the PEI backing was chosen for several reasons, e.g., good thermal stability, good impedance match to the PVDF copolymer, and very low acoustic attenuation [33]. The deposition method will be denoted as an adhesive-free method, since it uses only means for promoting bonding between the involved materials [PEI, sputtered electrodes, and P(VDF-TrFE)], and no additional adhesive layers. The adhesive-free construction will eliminate problems imposed by additional adhesive layers, like impedance mismatch and increased attenuation. The negative effects from additional adhesive layers are believed to strongly increase with frequency, although systematic studies on this are absent in the literature.

The main purpose of this paper is to investigate numerical and experimental methods that are suitable for modelling unfocused transducer arrays. This includes measuring and/or simulating the ultrasonic pressure field generated by the unfocused transducer elements, both internally in the elastic and piezoelectric layers, and remotely in the water tank. For the latter field, it was also important to investigate the interference field between multiple elements. The built prototypes were analyzed both in terms of acoustic performance and spatial distributions of the acoustic pressure fields employing narrowband and wideband excitations. The measured ultrasonic pressure fields were also compared with numerical model-based pressure filed using the k-Wave software. Many of the proposed experimental and numerical methods that are applied here to a specific type of transducers are quite general and flexible, and might therefore be adapted to other types of transducer configurations.

## 2. Materials and Methods

**Figure 1:**
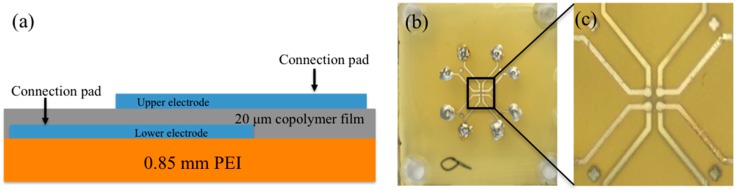
Schematic diagram of the adhesive free transducer (**a**), (**b**) the P(VDF-TrFE) unfocused transducer containing all four elements, (**c**) shows the magnified view of the figure (**b**).

Figure 1a, represents the schematic diagram of the transducer assembly. The first step involves plasma sputtering through a high-resolution mask, in order to produce a three-layer (Cr/Au/Cr) lower electrode on top of the PEI substrate of type Ultem 1000. Here, Cr was chosen to yield good bonding to materials on both sides of the electrode. Then, P(VDF-TrFE) in the fluid phase was spin coated on top of the lower electrode, and thereafter, dried to remove the solvent. A dried P(VDF-TrFE) thickness ~20 μm was achieved by adjusting the spin-coating parameters. Finally, a (Cr/Au/Cr) upper electrode was put on top of the dried P(VDF-TrFE) layer, which completed the transducer assembly process. Each prototype was designed to contain four active elements on a single PEI substrate, as shown by the overview image in Figure 1b, and magnified image in Figure 1c.

From Figure 1b,c the active elements can be identified as the four square-shaped domains towards the center of the image. The extension of each of these squares yielding the active areas was, under a microscope, estimated to be around 450 × 450 μm, while the element pitch (center-to-center) distance was estimated to around 1 mm. For each active area, two connection lines to the lower and upper electrodes can also easily be observed due to the transparency of the thin P(VDF-TrFE) layer. These lines provide electrical connections to a total of 8 connection areas (the outer areas in the Figure 1b with circular shape), which contains small pins drilled through the PEI substrate for further connection to a high voltage amplifier (lower electrode) and ground potential (upper electrode). For initiating the piezoelectric effect on the P(VDF-TrFE) thin layer, the transducers were polarized at room temperature. A high voltage AC source was connected to the lower electrodes, while the upper ones were connected to ground. Further details regarding the transducer production, annealing, and poling steps can be found in [34].

## 3. Experimental Setup for Acoustics Filed Pressure Measurement

**Figure 2:**
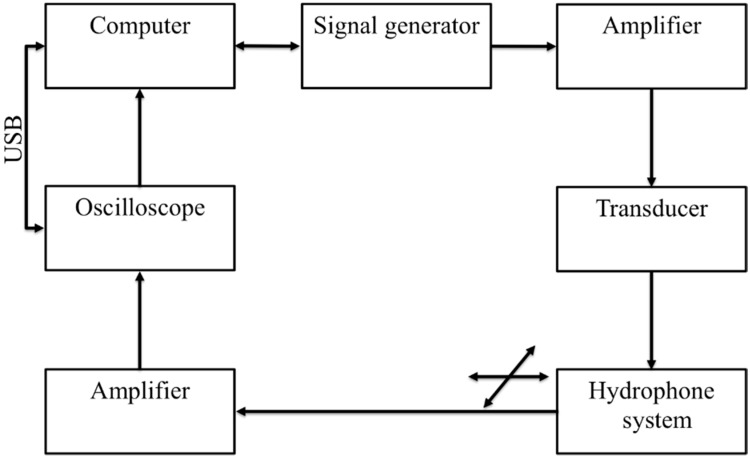
A schematic diagram of the ultrasonic fields measurement system. It consist of a signal generator that initiate the specified waveform which is scaled-up by an amplifier, and then loaded into the transducer. The emitted ultrasonic pressure from transducer was measured employing a hydrophone system including a booster amplifier. An oscilloscope then digitize the received signal and send it to a computer for software processing and storage. The oscilloscope and signal generator are parallelly connected with computer for synchronization of the scanning system.

In order to measure the ultrasonic field generated by the transducer prototypes, a hydrophone system was used (75 μm needle hydrophone) in combination with an ultrasonic scanning system [35]. For performing the ultrasonic scan, the transducer prototype was moved by a 3D actuation system, so that the ultrasonic field in planes parallel with the PEI plane (hereafter defined as the xy-plane) could be measured by the stationary needle hydrophone. By repeating this scanning procedure for planes at several distances from the scanning plane, a 3D view of the ultrasonic field can be constructed. An overview of the individual components used in the scanning system is shown in Figure 2.

All these components include an arbitrary signal generator (Tektronix AFG 3102, Beaverton, OR, USA) used to generate the desired pulse form either narrowband or wideband signals on the driver side. An RF amplifier (Electronics & Innovation: 403LA, Rochester, NY, USA) was employed to amplify the pulse-form before it was fed into the PVDF transducer. On the receiver side, i.e., the side that handles the hydrophone which includes a 75 μm needle hydrophone with a 1–30 MHz bandwidth (precision acoustics) [35] with an internal amplifier (denotes as the hydrophone system), an additional booster amplifier, and an oscilloscope (Agilent 3024A) were used. The hydrophone was moving in the x and y directions. The received signals generated from the transducer were received by the hydrophone and delivered to the oscilloscope. The oscilloscope performs averaging 256 pulse shootings, digitizes the signal, and records it onto a personal computer (PC) via a USB connection. The PC also controls the scanning in the xy-plane (step lengths and ranges set to 25 μm and 2 mm for both directions), and the distances from this plane to the transducer plane (fixed to 2, 4, 6, and 8 mm, respectively).

## 4. Results and Discussion

### 4.1. Transducers Response Measurement

**Figure 3:**
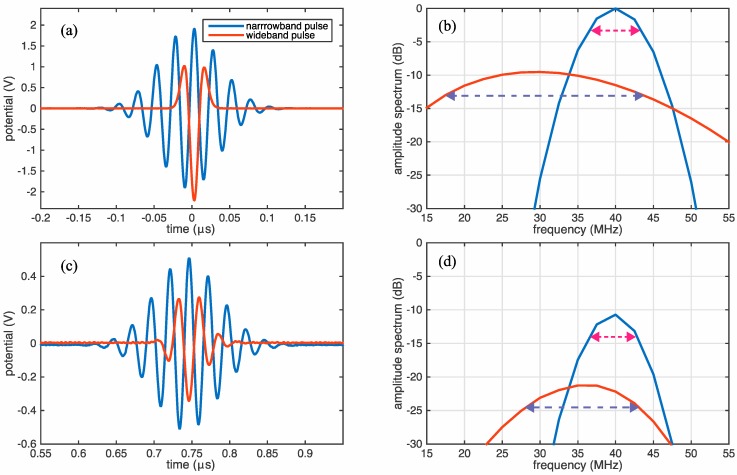
(**a**) Time-domain potential measurements for the narrowband and wideband pulses used to drive the transducer, and (**b**) their amplitude spectra. (**c**,**d**) Show the corresponding measurements for the waves reflected from the PEI backside, after amplifying the received current with a current amplifier.

The transducer characteristic response was first investigated by observing at the acoustic pulse reflections from the PEI backside using an experimental setup previously described in [21,22]. This setup uses an arbitrary signal generator (Agilent 81150A) that drives the transducer by applying the specified voltage to one of the transducer electrodes. The signal received on the counter-facing electrode is then amplified by a trans-impedance current amplifier (FEMTO DHPCA-100, Berlin, Germany), and digitized on an oscilloscope (Yokogawa DLM 6054, Tokyo, Japan) for further digital processing. For this test, both the narrow band and wide band pulses were applied with driving potentials measured as a function of time, as shown in Figure 3a.

The spectra corresponding to these pulses are shown in Figure 3b in a dB scale normalized to the maximum value for the narrowband pulse. From this figure, it is observable that the narrowband pulse has a center frequency of around 40 MHz, and a −3 dB bandwidth around 6 MHz. On the other hand, the wideband pulse has a slightly lower center frequency around 30 MHz, and a −3 dB bandwidth estimated to 26 MHz (see dotted arrows in Figure 3b). The corresponding measurements for the reflected pulse in the time and frequency domains are shown in Figure 3c,d, respectively.

From Figure 3b,d we notice that narrowband pulse has gained very small changes in the center frequency and bandwidth as it has propagated through the PEI backing. For the wideband pulse, it is noticeable that there is a slight upshift in the center frequency to around 36 MHz. We believe that this upshift is caused by the used current amplifier, which induces an amplification that is proportional to the wave frequency. The measurements also point out a reduction in the bandwidth for the wideband pulse, down to around 15 MHz. This reduction is probably mainly due to the amplitude enhancement imposed by the transducer around the central resonance. It is also likely that attenuation through the PEI polymer, and limitations in the bandwidth for the used instrument, have some impact on the reflection measurements.

### 4.2. Ultrasonic Field Imaging and Simulations

All hydrophone measurements were performed in double distilled water at room temperature (22 °C), where the bulk acoustic wave velocity of the water is estimated at 1488 m/s [36]. The measurements were also done with two different types of driver pulses. The first one, that we will denote as the narrow bandwidth pulse, is an amplitude modulated Gaussian pulse given by:

(1) $$V_{NB}=\;V_{0}\;\exp\left[-\frac{{\left(t-t_{0}\right)}^{2}}{2\sigma_{0}^{2}}\right]\sin\left[2\pi f_{0}\left(t-t_{0}\right)\right]$$

where $\sigma_{0}$ denotes the width of the Gaussian envelope centered at time $t_{0}$, while $f_{0}$ as the oscillation frequency. For the probe measurements and simulations presented in this section, the values, $\sigma_{0}=75$ ns and $f_{0}$ = 30 MHz were used to meet the upper bandwidth restrictions imposed by the hydrophone probe system. The second pulse, which we will refer to as the broad bandwidth pulse, is given by:

(2) $$V_{BB}=\;V_{0}\;\exp\left[-\frac{{\left(t-t_{0}\right)}^{2}}{2\sigma_{0}^{2}}\right]\left[1-\;\frac{{\left(t-t_{0}\right)}^{2}}{\sigma_{0}^{2}}\right]$$

with a width $\sigma_{0}=7.5$ ns. This pulse is also known as the Ricker wavelet.

Complementary to the ultrasonic measurements, numerical computations of the ultrasonic fields were performed using the commercial software COMSOL Multiphysics and the open source software package k-Wave. The first system (COMSOL Multiphysics), which is based on the finite element method (FEM), was developed and tested over twenty years, and applied within a large number of fields. Both the FEM method itself and the large number of solvers and grid generators provided with the system make it very suitable for implementing complicated geometries in different physical environments. It was therefore chosen to model the fabricated prototype transducer as a two-dimensional (2D), rotational symmetric model of piezoelectric PVDF film. The transducer electrodes, and the lower elastic PEI layer, are shown in Figure 3a. Since the layered transducer will typically be submerged in water as a coupling media during operation, an upper fluid layer has also been added to the FEM model, modeling the transducer water backing. 

**Figure 4:**
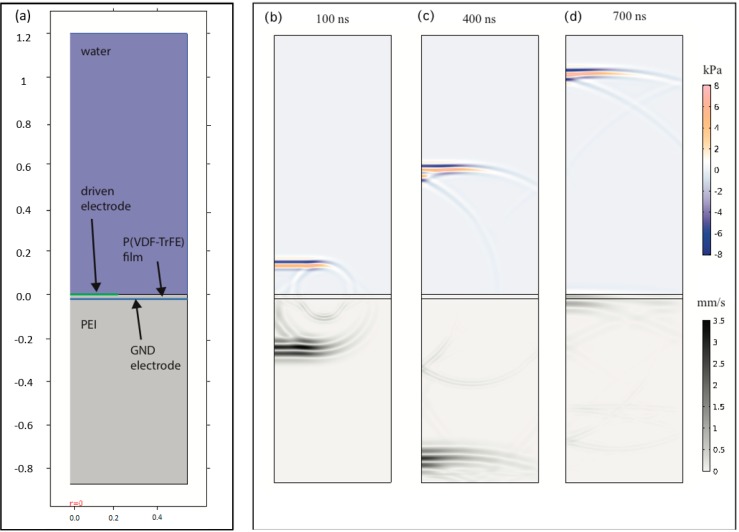
(**a**) Illustration of the computation domain and materials used in COMSOL with the locations of the driven and grounded electrodes shown by the green and blue lines, respectively. Computed pressures (shown in the upper water domain) and wave velocities (shown in the elastic domains) generated from a 0.45 mm electrode diameter of at times (**b**) 100 ns, (**c**) 400 ns, and (**d**) 700 ns after pulse firing.

An overview of the various domains used in the COMSOL model is shown in Figure 4a, with the locations of the driven and grounded electrodes shown by the green and blue lines, respectively. An example of wave fields produced from a driver electrode with diameter 0.45 mm at times 100, 400, and 700 ns after pulse firing, is shown in Figure 4b.

For this computation, the wideband pulse given by Equation (2) was applied, with parameters $\sigma_{0}=7.5$ ns (corresponding to a center frequency around 30 MHz) and $V_{0}$ = 2.3 V, which are typical for cases where the transducer is driven directly by a low voltage arbitrary signal generator (Tektronix AFG 3102). The obtained pressures (in unit KPa) are displaced in the upper water domain, while the norm of the displacement velocity (unit mm/s) is shown in the elastic PEI domain at the specified simulation times. The wave fields shown in Figure 4 is quite typical for fields exited from a P(VDF-TrFE) transducer with an aperture (driving electrode) that is significantly larger than the water and PEI wavelengths, e.g., with a strong planar longitudinal wave-front observed both on water and in the elastic PEI material. From Figure 4a, we notice that the longitudinal wave front in the PEI material, which is the fastest propagating wave feature, has become reflected from the lower boundary (modeling the PEI-water interface) just before 400 ns, and reaches the transducer film after around 700 ns. A slower propagating wave with circular shape is also observed, which arises from the end point of the driven electrode. This wave feature appears both as the surface of the material interfaces, and as a shear wave in the PEI domain.

To model 3D propagation of waves into the water tank underneath the transducer and wave coupling between several transducer elements, a second software package, known as k-Wave, was preferred. The k-Wave software is an open-source MATLAB toolbox for modelling ultrasonic waves in compressible media (gaseous and fluids) or in elastic materials. Some of the time-critical functions are provided as pre-compiled binaries for running on central processing units (CPUs) or graphics processing units (GPUs). The software is based on a so-called *Fourier collocation spectral method*, with the solution expanded in terms of a global Fourier series. The global nature of the Fourier basis has both benefits and weaknesses compared to, for example, COMSOL Multiphysics, which, through the FEM approach, uses both the basis and weight functions with a local nature. A fast computation time and very high accuracy can be achieved with k-Wave, both of which are very beneficial in large-volume 3D computations. However, the global basis restricts the computational domain to rectangular areas (2D) or volumes (3D), and also constrains the geometry for the source and receiver elements. The k-Wave software also requires sufficient smoothness for the obtained solutions, which can typically be achieved by using low-pass filters implemented in the package or smoothed wave sources.

Despite the aforementioned limitations of the k-Wave page, we found it very useful for computing 3D wave fields in water. The main reason for this is its faster computational time, compared to COMSOL; this was needed to model the 3D interference and diffraction patterns produced by several transducers, typically requiring a large water domain (measured in terms of the water wavelength). The k-Wave achieves high speeds by using a collocation method in the Fourier domain, and has the ability to do parallel processing on both CPUs and GPUs. Since, k-Wave works in the Fourier domain, it is also straightforward to implement periodic boundary conditions, which again, makes it easy to model wave-interference between the transducer elements presented here. Unlike the used COMSOL version (5.2 A), k-Wave has pre-implemented absorbing boundary conditions in the time domain, yielding very small reflections as the waves reach the boundaries. This is very convenient for large water tanks, which can be approximated as an infinitely large volume using absorbing boundaries. It should be noted that k-Wave has much less flexibility than COMSOL when it comes to geometries and physical models. The water tank, therefore, has to be modeled as a rectangular 3D tank, and since k-Wave cannot simulate elastic or piezoelectric materials, the wave field produced by the transducer and the PEI domain has to be simplified as a time-domain source inside the tank.

**Figure 5:**
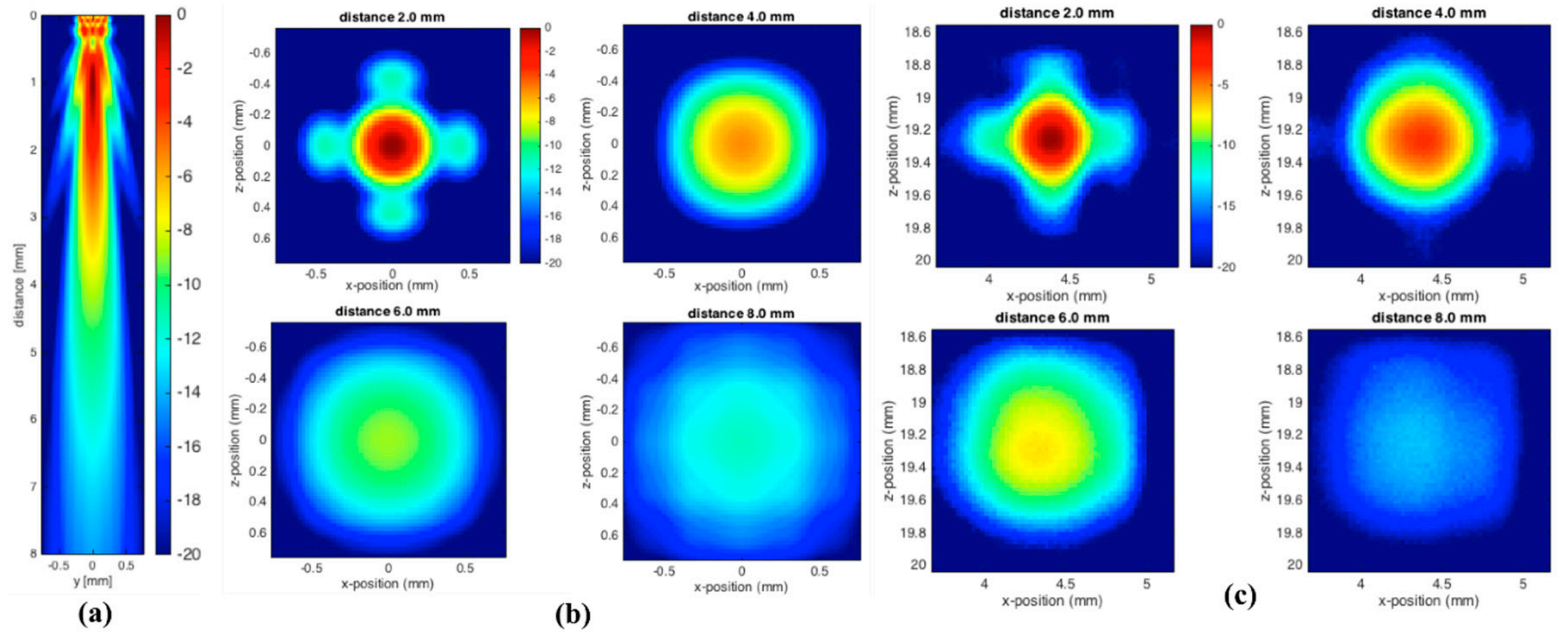
(**a**) Simulated ultrasonic pressure amplitudes (k-Wave results in the xz-plane) obtained for a single transducer element driven by the narrow bandwidth pulse; (**b**) numerical simulation of ultrasonic pressure field in the xy-plane at 2, 4, 6, and 8 mm; and (**c**) hydrophone measurements of a single transducer applying a narrowband Gaussian modulated signal at different distances, such as 2, 4, 6, and 8 mm, with respect to hydrophone.

Figure 5 shows the ultrasonic pressure amplitudes obtained for a single transducer element driven by the narrow bandwidth pulse $V_{NB}$. The sub-figures presented here, include both the k-Wave results in the xz-plane Figure 5a and in the xy-plane Figure 5b, and the hydrophone measurements in the xy-plane Figure 5c.

As expected, the highest pressure-amplitudes were observed around the center of the electrode, both for the measurements and the numerical model with a slight offset for the measurements. The side view obtained from the numerical model [Figure 5a] clearly shows that the pressure field consists of a main-lobe (central pressure distributions) and a 1st and 2nd side-lobe that propagates outward on both sides. The contribution from the 1st side lobe can also be identified in both Figure 5b,c at the distance closest to the transducer (2 mm) as the four outgoing arms around the central lobe. At distances further increased from the transducer plane, the side-lobes become invisible except for at 4 mm in Figure 5c, where they are barely visible but present nonetheless. This result was expected since side lobes will decay faster than the main lobe as the distance increases from the transducer plane. These side lobes can also be described with the aid of the diffraction effect from the apertures [37]. The diffraction effect is a common phenomenon in optics and other fields involving waves. In terms of wave propagation, each point on a wave front may be assumed as a source of a spherical point source. These sources interfere with each other and form a new wave front yielding a diffraction pattern with high and low-pressure areas. In particular, for a monochromatic wave source, we expect to find several low-pressure areas, given the analytic expression:

(3) $$\sin\left(\alpha \right)=\frac{\pm N\lambda }{w}$$

where N is a natural number (N = 1, 2, …). In this formula α is the quiet zone angle with respect to the transducer plane normal vector, λ is the wavelength, and w is the common width of the square transducer aperture. If we assume that the center frequency $f_{0}=30$ MHz is the dominant feature for the narrow bandwidth pulse, then Equation (1) yields a first low-pressure zone with an opening angle α≃9.6° assuming N=1, λ=75 μm and w=450 μm. This produces, for example, a circular low-pressure region with radius ≃0.34 mm at a distance 2.0 mm from the transducer. This estimate matches well with Figure 5b, taken at 2.0 mm, since the low-pressure circle will appear between the main lobe and the four side lopes observed toward the edges in the image. By comparing Figure 5b,c, we also notice rather good agreement between the experimental and numerical findings, especially at the closest distances, where rather similar diffraction patterns can be observed. However, the measured pressure fields seem to be more smoothed than the numerical ones, which could be due to both noises in the measurements and deviation from the assumed rectangular shape in the numerical model. It is also likely that the size of the used hydrophone needle contributed to the observed smoothing of the experimental data. 

Later on, the acoustic field pressures were also imaged in an increasing manner (2, 4, 6, and 8 mm) with respect to the excited transducer plane and receiving hydrophone. In the near field, the acoustic pressures were intense, while in the far field the intensity diminished, due to week acoustic field pressure. Later on, the experimental procedure was performed with wideband driver pulse $V_{WB}$, in order to compare to the narrowband results.

**Figure 6:**
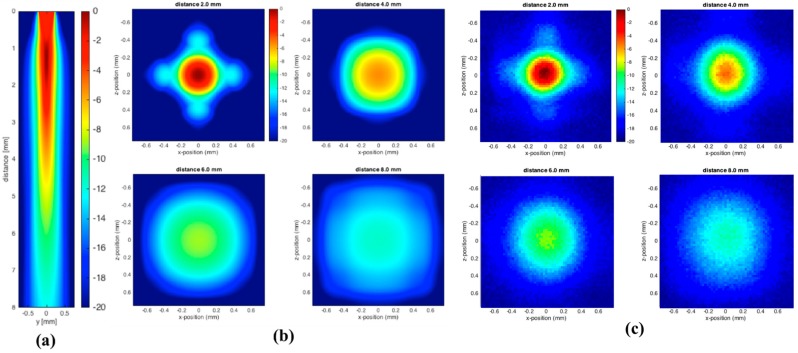
(**a**) Simulated ultrasonic pressure amplitudes (k-Wave results in the xz-plane) obtained for a single transducer element driven by the broad bandwidth pulse, (**b**) numerical simulation of ultrasonic pressure field in the xy-plane at 2, 4, 6, and 8 mm, and (**c**) hydrophone measurements of a single transducer applying a Gaussian 2nd derivative (wideband) signal at different distances such as 2, 4, 6, and 8 mm with respect to hydrophone.

From Figure 6, which summarizes both the numerical and experimental results, we notice that the side lobes have diminished, e.g., by comparing side views Figure 5a and Figure 6a. This is likely due to the much broader bandwidth of $V_{WB}$ that will smear side lobes, since their angular distribution is frequency-dependent, as predicted by Equation (3). However, it noticeable from Figure 5b that the scans taken closest to the transducer (2.0 and 4.0 mm) demonstrate some extension of the pressure field along the x- and z-axis, which is probably the remains of the side lobes. Also, for the broad-banded pulse, the experimental and numerical results match quite well.

## 5. Conclusions

In this paper, we have explored the characteristic responses and acoustic field distributions of unfocused high frequency array transducers. A transducer array made from an adhesive free method was investigated using suitable experimental and numerical means for two different driving pulses (narrow and wide band). Reflections induced from these driving pulses were then used to estimate the transducer sensitivity and bandwidth. Later on, the acoustic pressure distributions were measured using a hydrophone, and imaged at different distances from the transducer surface, both for the narrow- and wide-band pulses. The experimental results were also compared to a 3D numerical model using the k-Wave software, yielding consistent results and comparable wave field distributions at the investigated transducer distances. Unfocused transducer arrays like the one presented in the current manuscripts have applications for many fields within medicine and non-destructive testing. This includes both near-field imaging, where single elements are typically activated or scanned electronically, and far-field imaging, using, for example, electronic steering of several elements.
